# Resource-aware video streaming (RAViS) framework for object detection system using deep learning algorithm

**DOI:** 10.1016/j.mex.2023.102285

**Published:** 2023-07-15

**Authors:** Ary Mazharuddin Shiddiqi, Edo Dwi Yogatama, Dini Adni Navastara

**Affiliations:** Department of Informatics, Institute Teknologi Sepuluh Nopember, Indonesia

**Keywords:** Video stream mining, Resource-aware streaming framework, Deep learning, Resource-Aware Video Streaming (RAViS) Framework

## Abstract

Video streams can come from various sources, such as surveillance cameras, live events, drones, and video-sharing platforms. Video stream mining is challenging due to the extensive resources needed to analyze and extract useful information from continuous video data streams. This situation could result in overwhelmed resources, which causes the system to stall. One of the ways to suffice the requirement is to provide larger resources, which leads to more costs. This research develops a data stream mining called the Resource-Aware Video Streaming (RAViS) framework to adapt to the limited resources (a Raspberry Pi) to run an object detection system using the YOLO algorithm. We validate the framework by capturing video streaming to simulate data streams. The video frames are processed using a deep-learning model to recognize the presence of a person(s) in a room. The RAViS framework adapts the object detection system to the availability of Raspberry Pi resources, such as CPU, RAM, and internal storage. The adaptation aims to increase the availability of resources to perform object detection of streamed video. The experimental results indicate that the RAViS framework can adapt the detection system to resource availability while maintaining accuracy.

• A framework can ensure the availability of a computer with limited resources for running an object detection system using deep learning algorithms.
• The framework constantly monitors the computer's memory, CPU, and storage, and provides feedback to the object detection system for adjusting its parameters to optimize resource utilization.
• This approach enables the object detection system to operate continuously with the required resources, thus ensuring its accuracy and effectiveness.

## Graphical abstract

**Figure:**
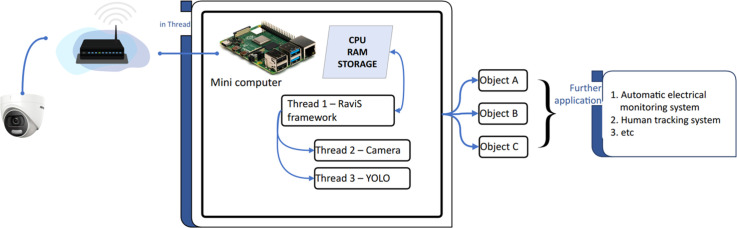

Specifications table

**Table:** 

Subject area:	Computer Science
More specific subject area:	Data Stream Mining
Name of your method:	Resource-Aware Video Streaming (RAViS) Framework
Name and reference of original method:	J. Redmon, S. K. Divvala, R. B. Girshick and A. Farhadi, “You Only Look Once: Unified, Real-Time Object Detection,” in Proceedings of the IEEE Conference on Computer Vision and Pattern Recognition (CVPR), Las Vegas, NV, USA, 2016.
Resource availability:	N/A

## Method details

 

## Introduction

A data stream is a continuous flow of data generated, processed, and consumed over time. Data streams can be generated from various sources, such as sensors, network devices, social media, and other applications producing large data volumes. The analysis process for such data needs to be real-time to detect any data deviations. Thus, the related system can act fast if anything is found. Examples of data stream processing include IoT sensors for environmental monitoring to capture earthquake symptoms, potential landslides, etc. Processing such data requires high computational resources (e.g. memory, CPU, and disc space). It may sometimes lead to resource scarcity if not properly managed. The possibility of a system experiencing resource-scarce can be even worse for processing video streams—for example, employing cctv for object detection or surveillance. Therefore, it is necessary to manage resource usage to suffice the need and maintain computation performance.

Many research have been conducted to manage resources to process data streams in real time properly. Referring to research by Gaber and Yu [1], three settings are used to manage resource availability during the data stream mining process, i.e. input, process, and output. The input management is named Algorithm Input Granularity (AIG), the process management is named Algorithm Process Granularity (APG), and the output management is named Algorithm Output Granularity (AOG). This three-granularity management can be called Algorithm Granularity Settings (AGS). The research has shown that the AGS can preserve resources while processing data streams.

The rise of high data volume, particularly video, presents a significant challenge for resource-aware frameworks. This challenge becomes even more complex when resource-intensive processes, such as object detection using deep learning algorithms, are involved. To address this issue, some researchers have proposed compression techniques that can adapt to low-bandwidth resources by adjusting their compression based on the video characteristics and network bandwidth [2,3]. However, studies have shown that there is a trade-off between the accuracy of object detection and the compression technique used [4], [5], [6]. Another issue is the processing speed as addressed in [7,8] where video analytics for object detection and classification process requires compute-intensive GPU-powered servers in the cloud. Research in [9] have shown the need to consider both algorithm and hardware resource aspects for object detection, considering. These findings have highlighted the need for further research in this area. To best our knowledge, there is still limited study in developing a resource-aware framework for video stream mining.

We develop a novel framework called Resource-Aware Video Streaming (RAViS) to address the challenges posed by high data volume and resource-intensive processes in video stream mining. To the best of our knowledge, this framework represents the first of its kind in addressing resource-awareness for maintaining resource availability and optimizing performance in object detection processes. Our research contributes to this area by developing a resource-aware framework that maintains the availability of computer resources while ensuring object detection accuracy. We have elaborated the framework by incorporating a deep learning algorithm for object detection, enhancing its capabilities. The contribution of our research are:

• develops a resource-aware framework to maintain the availability of computer resources.
• elaborates the framework with a deep learning algorithm for object detection.
• maintains the accuracy of object detection when adjustments are performed based on the available resources.

The rest of the paper presents the literature review in Section II, the proposed method in Section III, the experimental results in Section IV, discussions in Section V and to finally concludes our study in Section VI.

## Literature review

We present background and related works to our proposed method in this section, i.e. data stream mining, resource-aware framework and deep learning algorithm. At the end of this section, we present the state of the art of research in this field.

### Data stream mining

**Fig. 1:**
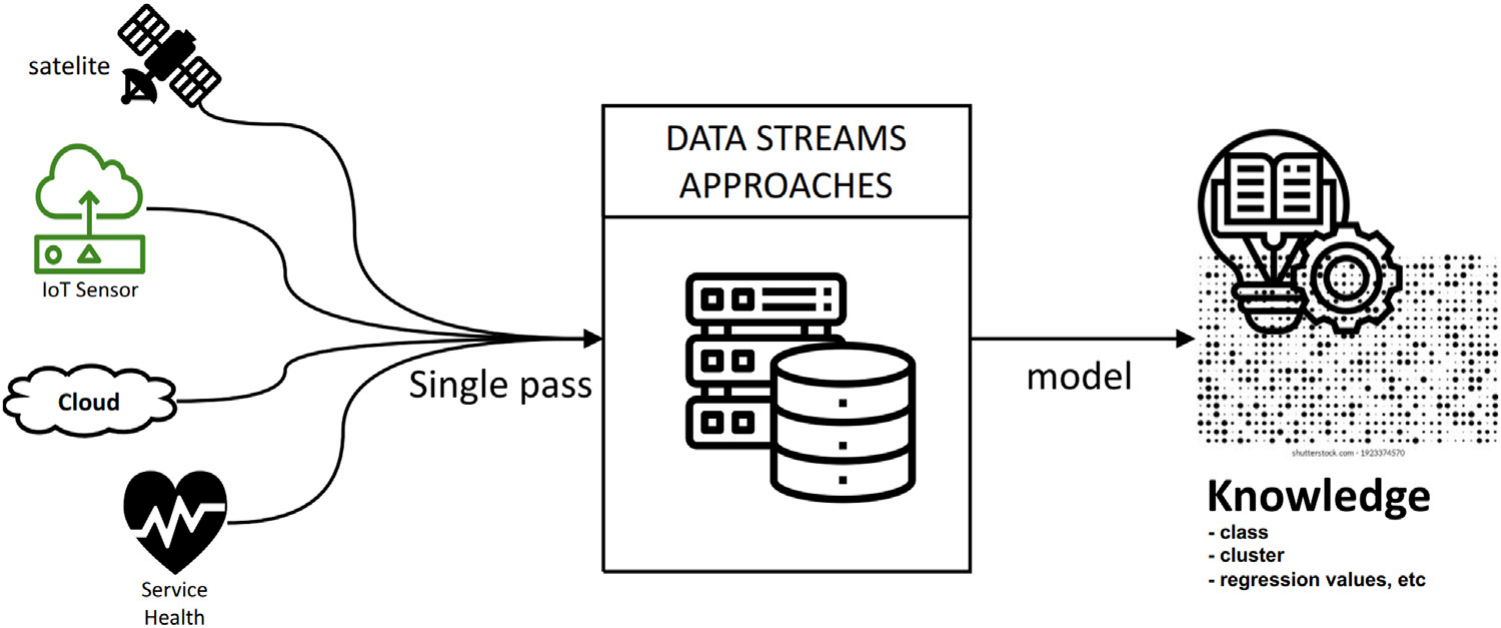
The architecture of data stream mining.

Mining data streams have attracted many research due to emerging embedded technologies [1]. Data stream mining aims to extract knowledge from continuous data records entering a system (Fig. 1). According to research in [10], data streams can be interpreted as a series of sequential data in real-time that comes continuously over time. Real-time data stream mining can help businesses develop strategies, advance knowledge, and support research in science and medicine by identifying essential data patterns [11,12] or context [13].

Data streams are simply information data that continuously flows. There are two types of data streams: transactional and measurement data streams [10]. Transactional data streams record interactions between data attributes, such as purchases or telephone data. Measuring data streams monitor changes in entity status from sensor data. For example, predicting weather and traffic trends based on historical weather and traffic data.

#### Resource-aware framework

Software systems have become more resource-intensive, often exceeding hardware capabilities and resulting in resource scarcity issues. To avoid this issue, software systems must be “resource-aware” and match their computing needs to the state of hardware resources [14]. A resource-aware framework allows users to monitor computing resource usage and implement policies for resource management. In cases of resource overuse, the program's hierarchical structure enables asynchronous notifications and computation termination [15].

#### Algorithm granularity settings

In the context of the resource-aware framework, Gaber & Yu developed an Algorithm Granularity Settings (AGS) to adjust an application's input, output, and process based on current resource conditions [1]. The AGS monitors resource consumption over time to manage computing resources such as memory, processor, and battery. Periodic or aperiodic adjustments are made based on resource availability changes. Periodic adjustments are for gradual changes in resource availability, while aperiodic adjustments respond to significant changes in resource availability. The AGS comprises three components:

• Algorithm Input Granularity (AIG) adjusts the speed of the incoming data stream to the Stream Mining algorithm, with techniques such as sampling, load shedding, and synopsis of the data.
• Algorithm Output Granularity (AOG) alters the magnitude of the algorithm's output to meet memory limitations, with lower granularity values for more detailed output requiring greater memory capacity.
• Algorithm Processing Granularity (APG) changes computation parameters to maintain computational resources (CPU).

The AGS observes resource consumption requirements statistically over time and changes parameters in the AIG, AOG, or APG according to the output of resource monitoring. Then, the mining algorithm adapts to the settings changes the AGS performs.

### Deep learning

The performance of machine learning algorithms heavily relies on the quality of input data representation. However, the need to transform data with a high feature level into a lower feature representation before being processed by machine learning algorithms can be a drawback. In contrast, Deep Learning can handle large datasets and is able to learn complex representations of raw data, including images and text.

Deep Learning has gained increasing attention due to its ability to process large and complex data sets. With the rapid development of hardware technology, such as High-Performance Computing (HPC) and Graphics Processing Units (GPUs), and the availability of large and diverse datasets, Deep Learning has become a powerful tool for solving complex problems in various fields [16,17]. Consequently, there has been a surge in research on Deep Learning and Distributed Learning to explore their potential in various applications.

Deep Learning uses supervised and/or unsupervised strategies to learn hierarchical features that automatically extract information from raw data using the structure of hidden features [17]. Deep Learning builds multi-layer learning models using transformations and graphs. One of the most popular Deep Learning algorithms is Convolutional Neural Networks (CNN), which can automatically identify irrelevant features without human information [16]. Unlike traditional fully connected networks, CNN employs shared weights and local connections to process input data with a two-dimensional structure, such as images.

### You only look once (YOLO) algorithm

The YOLO algorithm is utilized to detect objects in images based on regression problems to produce object class probabilities [18], [19], [20]. The algorithm offers a balanced detection speed and accuracy. The latest YOLO architecture includes the CSPDarknet as the backbone, the PANet as the neck, and the YOLO layer as the head. This architecture delivers improved performance compared to previous versions [21]. The YOLO regression-based technique centralizes image detection targets and feature extraction processes. It does not require segmentation to produce detection results, reducing the detection process's computational cost [22]. However, the YOLO algorithm may need to improve the detection of small objects since the grid sizes may lead to multiple targets in one grid [23,24]. Studies have shown that the YOLO algorithm provides promising results and may become increasingly useful for object detection.

#### Tiny yolo

Tiny YOLO is a faster and smaller version of the YOLO algorithm for object detection. It is about 442% faster than the native YOLO and achieves 244 FPS on a single GPU, but at the cost of lower accuracy. Compared to the native YOLO's accuracy range of 51–57% on the COCO dataset, the accuracy of Tiny YOLO is only 23% [25]. However, newer versions of Tiny YOLO, such as Tiny-YOLOv4, have been developed to improve accuracy and speed performance. The architecture of Tiny-YOLOv4 includes a backbone network, neck, and head. The backbone network is based on the CSPDarknet53-tiny structure, while the neck adopts the Feature Pyramid Network (FPN) structure to integrate features of different scales. The input size is 416 × 416, and multi-scale detection is performed by combining the idea of FPN and two detection layers as output. The two feeding detection layers divide the input image into 26 × 26 and 13 × 13, respectively, with each grid cell predicting three bounding boxes. Each bounding box contains five elements: x, y, w, h, and Ci, with x and y representing length and width after normalization proportional to w and h, while Ci is the confidence score.

#### Resource consumption

A study in [26] compared the performance of tiny YOLO on various platforms. The study used multiple devices, including Jetson Nano, Jetson TX2, Jetson Xavier NX, and self-built devices. The results revealed that the tiny-YOLO inference model process required significant memory usage even with GPU-equipped devices. For instance, Jetson Nano required 966 MB of memory, Jetson TX2 required 1044 MB of memory, and Jetson Xavier NX required 1265 MB of memory. These findings suggest that CNN implementation demands considerable resource consumption depending on memory capacity and run-time bandwidth. A similar study [27] compares the CPU utilization, GPU utilization, object accuracy, latency, and power consumption of the Nvidia Jetson AGX Xavier platform frameworks. The study suggests that the Jetson platform is efficient for developing deep learning-related object detection technology.

### Raspberry Pi dan raspbian Os

The Raspberry Pi is a single-board computer that has gained popularity in IoT, real-time image/video processing, and robotic applications. Its architecture includes a 40-pin GPIO connecting various modules such as LEDs, motors, and sensors. It features an ARM-based processor with CPU speeds ranging from 700 MHz to 1.2 GHz and onboard SDRAM capacities ranging from 256 MB to 1 GB. The Raspberry Pi also includes SPI, I2C, I2S, and UART modules. Some models have a GPU to speed up image processing calculation. Several operating systems support Raspberry Pi, including Linux-based systems like NOOBS OS, Ubuntu, Archlinux RISC OS, and Windows 10 IoT Core. One commonly used operating system is Raspbian OS, a free and open-source operating system based on Debian Linux. The Raspberry Pi includes a user-friendly GUI and tools like browsers and Python. Pi Foundation released Raspberry Pi in 2012 to facilitate programming and hardware projects, home automation, and industrial applications.

One of the most popular uses of the Raspberry Pi is for real-time image and video processing, thanks to its powerful processing capabilities and GPU support. The Raspberry Pi can be used for object detection and recognition, facial recognition, and even autonomous vehicle navigation. It can also be used in robotics applications, such as controlling motors and sensors.

### State of the art

Resource-aware frameworks have typically been developed for systems with limited resources such as sensor networks. However, devices with higher resource specifications that run tasks beyond their capabilities can also face resource scarcity. For example, running an object detection system on the Raspberry Pi using the YOLO algorithm requires significant computation, making it necessary to have a resource-aware framework to manage resource allocation efficiently. These techniques help to ensure that the detection system performs effectively without causing resource scarcity or excessive energy consumption. Therefore, our proposed RAViS framework plays a vital role in ensuring the availability of resources required by a system to operate effectively.

## Proposed method

**Fig. 2:**
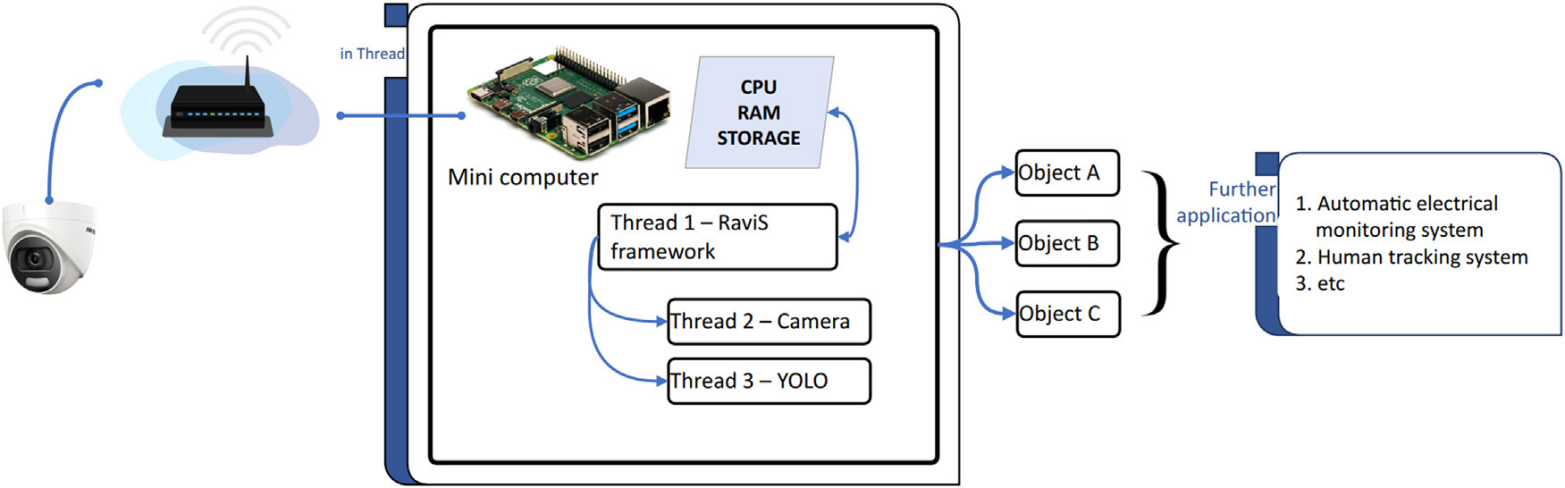
System architecture.

The proposed object detection and surveillance system includes a Resource-Aware Video Streaming (RAViS) framework that monitors the CPU, memory and storage conditions of the Raspberry Pi 4. This system disregards battery usage since the device is connected to electricity in a building. The challenge in designing the RAViS framework lies in maintaining resource availability while preserving object detection's performance without significant degradation. This design necessitates the identification of specific parameters or variables within the object detection algorithm that need to be adjusted to accommodate changes in resource availability. Our proposed system comprises a camera and a minicomputer (Raspberry Pi 4), responsible for retrieving and performing object detection tasks (Fig. 2). Resource adaptations are performed based on the consumption of the object detection system.

The camera streams images to the Raspberry Pi 4, which processes them to detect the presence of humans in a room. The YOLO algorithm is used to detect the objects, and the detection results, including the objects' names, are stored in a file. A predetermined threshold is set to determine the output of the process. If the detection value is below the threshold, the output is 0, but if it is above the threshold, the output is 1. The images captured from surveillance video frames are sent to a server for analysis using a deep learning model to detect the objects' presence in a room. Once the objects are identified, further actions can be taken, such as turning on/off the electricity for appliances in the room.

### The main thread

The main thread acts as a communication bridge between the running threads while monitoring feedback from the RAViS framework to manage the entire process (Algorithm 1). The program begins by starting all threads: RAViS framework to monitor resources, stream video from the camera, and perform object detection using the YOLO algorithm.

**Algorithm 1:** Main thread.

1: Start all thread()
2: while TRUE do
3: Camera.stream()
4: Camera.setTimeCapture(ravis.getTimeCapture())
5: Yolo.setTimeout(ravis.getTimeout())
6:
7: // RAViS framework feedback on RAM
8: if (RAM warning:) then
9: Restart()
10: end if
11:
12: // RAViS framework feedback on CPU
13: if (CPU warning:) then
14: Yolo.setTimeout(10)
15: end if
16:
17: // RAViS framework feedback on Storage
18: if (Storage warning:) then
19: Free space
20: Sleep(5)
21: end if
22:
23: if (yolo.result is not None:) then
24: if (yolo.result > 0 and yolo.result < 2:) then
25: Action is taken
26: else if (yolo.result ≤ 0:) then
27: Action is not taken
28: end if
29: end if
30: end while

The RAViS framework is run as a separate thread from the object detection thread to avoid interference. This system enables the object detection process to run smoothly without causing resource scarcity, ensuring optimal system performance. The RAViS framework reports system resource usage. If there is a RAM warning, the program restarts. If there is a CPU warning, the program sets the timeout to 10. If there is a storage warning, the program frees up space and sleeps for 5 s. Finally, if the YOLO object detection system returns a result, the program takes action based on the result. An action is taken if the result is greater than 0 and less than 2, which means that the YOLO has successfully detected an intended object. No action is taken if the result is less than or equal to 0.

### Resource monitoring threads

Each resource monitoring component runs on a separate thread, which operates similarly to the main thread. The threads periodically check the RAM, CPU, and storage usage (Algorithm 2).

**Algorithm 2:** The RAViS framework.

Input: ram = monitorRAMPrecentage()
Input: cpu = monitorCPUPrecentage()
1:
2: while TRUE do
3: // PROCEDURE WatchRAM
4: if (ram > *τ* RAM and ram < 100) then
5: ramWarning = FALSE
6: tcapture =1 + (1 - *τ*RAM) × cRAM
7: cRAM = cRAM + 1
8: else if (ram >= 100:) then
9: ramWarning = TRUE
10: else
11: cRAM = 1
12: ramWarning = FALSE
13: end if
14:
15: // PROCEDURE WatchCPU:
16: if (cpu > *τ* CPU and cpu < 100:) then
17: cpuWarning = FALSE
18: tprocess = randomBetween(1 − 5) × cCPU
19: cCPU = cCPU + 1
20: else if (cpu >= 100:) then
21: cpuWarning = TRUE
22: else
23: cpuWarning = FALSE
24: cCPU = 1
25: end if
26:
27: // PROCEDURE WatchStorage:
28: if (storage > *τ* Storage and storage < 100:) then
29: storageWarning = FALSE
30: treset =1 + (1 - *τ*Storage) × cStorage
31: cStorage = cStorage + 1
32: else if (storage >= 100:) then
33: storageWarning = TRUE
34: else
35: storageWarning = FALSE
36: cStorage = 1
37: end if
38: end while

Running each component on a separate thread allows for efficient parallel processing, which improves the system's overall performance to handle a larger volume of data. However, running multiple threads concurrently also increases the system's resource usage. To ensure that the system does not become overloaded, each thread performs periodic resource usage checking and adjusts its resource consumption accordingly. This helps to prevent the system from becoming unresponsive or crashing due to excessive resource usage.

#### RAM

The RAM monitoring module aims to ensure the availability of RAM to run the object detection system. This module consists of several procedures:

• let M be the total amount of memory available in the system. Then *m*_1_ + *m*_2_ + … + *m*_n_ <= *M* where [1,*n*] is the memory allocated to each task running in a machine.
• if the RAM usage has reached 100%, then the image-capturing process will be stopped temporarily to execute commands to free up RAM, such as removing the program cache
• a threshold value (*τRAM*) is used as the lower bound of memory usage. If the resource usage is less than *τRAM*, then the RAM usage counter (*сRAM*) is reset to one
• if the RAM usage is between *τRAM* and 100% then the image capturing interval (*t*_capture_) is calculated using Eq. (1).
  (1) $$t_{capture}=1+\left(1-\tau RAM\right)\;\times cRAM$$
  *Adjusting the time to capture images based on RAM availability.*

**Fig. 3:**
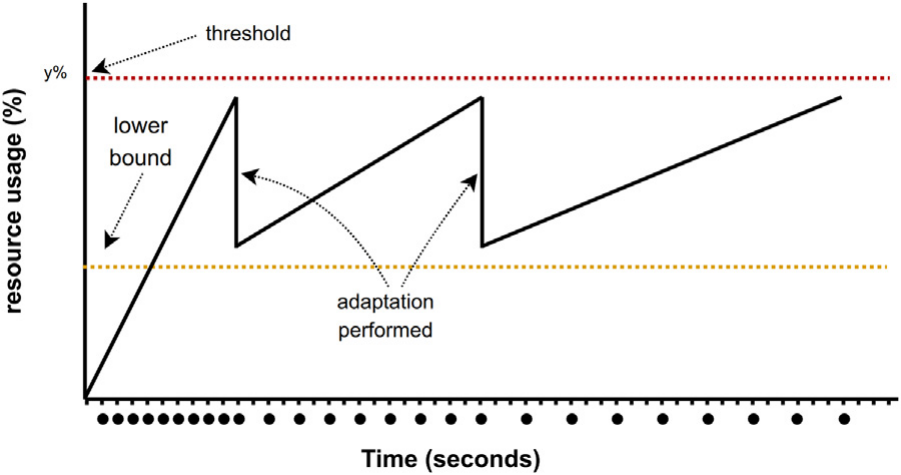
The RAM and storage adaptation mechanism is performed by adjusting the image-capturing and reset frequency.

The illustration of RAM perspective monitoring can be seen in Fig. 3. The dots are times when image-capturing is performed. Initially, image-capturing is performed once every second. However, this can be changed to fit the RAM usage. The figure shows adjustments are made when RAM adjustment procedures are met. In this case, the image-capturing intervals are getting longer, resulting in fewer images kept in the RAM. As a result, the availability of RAM is maintained.

#### CPU

**Fig. 4:**
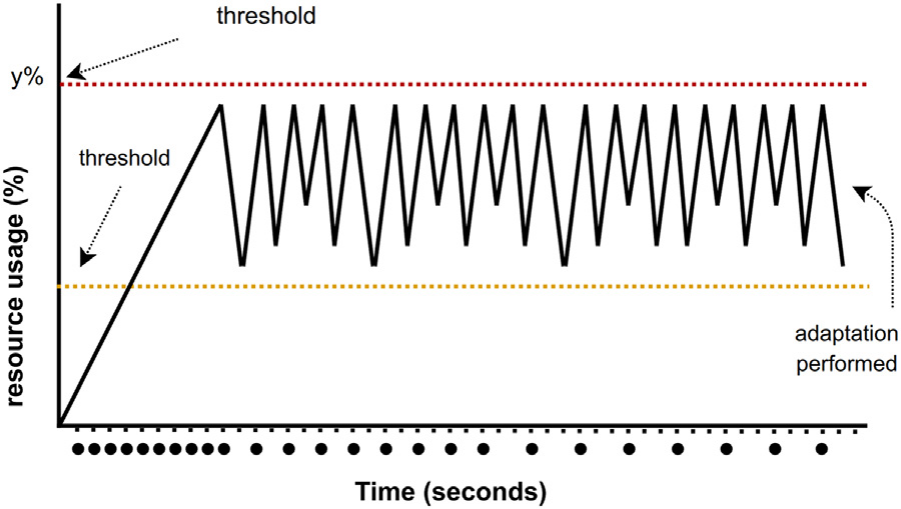
The CPU adaptation mechanism is performed by adjusting the image processing for object detection frequency.

Despite the Raspberry Pi's tiny YOLOv4 being a fast option for image processing, a growing data queue can increase CPU load. Therefore, procedures for CPU monitoring are necessary. The CPU monitoring module observes image processing tasks for images stored in memory and adjusts detection system parameters accordingly (Fig. 4). The procedures to perform CPU monitoring are:

• Let *C* be the total number of CPU cycles available in the system. Then *c*_1_ + *c*_2_ + … + *c*_n_ <= *C* where [1, *n*] is the CPU allocated to each task running in a machine.
• if the CPU usage reaches 100% then the image detection process will be stopped for 10 s
• a threshold value (*τCPU*) is used as the lower bound of CPU usage. If the CPU usage is less than *τCPU* then the detection process continues to run, and the CPU usage counter (*cCPU*) is reset to one.
• if the CPU usage is between *τCPU* and 100% then the CPU stops the detection process temporarily (*t*_process_) based on Eq. (2).
  (2) $$t_{process}=\text{randomBetween}\left(1-5\right)\;\times cCPU$$
  *Adjusting the time to process images based on CPU availability.*

The detection output is the average score derived from the detection results of multiple images. This approach minimizes error detection arising from perturbed images. When CPU adjustments are performed, the results of the object-detecting process might be impacted. The RAViS framework prompts the Yolo thread to pause for a few seconds when CPU usage is high. The outcome of this action is fewer images processed by the Yolo algorithm. Since fewer images are processed, the average detection score could be lower due to fewer images that can compensate for detection failure if perturbed images are present.

#### Storage

The storage monitoring procedures are mainly similar to the RAM monitoring (aims to maintain the availability of storage). Since the procedures of the storage adjustments are quite similar to the RAM adjustment, the expected adaptation results will be similar to the illustration in Fig. 3. The details of the procedures are:

• Let S be the total number of storage available in the system. Then *s*_1_ + *s*_2_ + … + *s*_n_ <= *S* where [1,*n*] is the storage allocated to each task running in a machine.
• If the storage usage has reached 100% then the system deletes old data.
• A threshold value (*τStorage*) is used as the lower bound of storage usage. If the storage usage is less than *τStorage* then the storage usage counter (*cStorage*) is reset to one.
• If the storage usage is between *τStorage* and 100% then the image-capturing interval (*t*_reset_) is calculated using Eq. (3).
  (3) $$t_{reset}=1+\left(1-\tau Storage\right)\;\times cStorage$$
  *Adjusting the time to process images based on CPU availability.*

To prevent excessive adjustments by the RAViS framework, limitations are applied. This limitation ensures that the detection process can continue to acquire data for the detection process without interruption. Image-capturing and storage reset are limited to a 5-second interval, and detection processes are limited to a 20-second interval. This mechanism balances efficient processing and storage management to achieve optimal performance.

### Capturing images mechanism

The system stores the captured images in the storage. The images are saved before the object detection process commences to enable efficient processing. This approach is preferred over applying object detection on a video stream directly since it is computationally heavier. The saved images are used for tracking the data processed by object detection. However, this approach can cause full storage, preventing the system from saving additional images. Let *N* be the number of cameras running in the system, *I* be the size of each image file in bytes, and *S* be the maximum storage capacity of the system in bytes. Then we can say that the required storage (*reqStorage*) per batch capture equals *N* x *I*. The time until the storage runs out (*t*_storageOut_) can be calculated using Eq. (4). To circumvent this issue, the system performs an adjustment process by slowing the image capturing for a certain period before the storage runs out, thereby extending its run-time.

(4) $$t_{storageOut}=\left(S-reqStorage\right)/\left(N\times I/t\right)$$

*Adjusting the time to process images based on CPU availability.*

The system incorporates multiple cameras running on a separate thread for video streaming. Upon capturing a frame, the thread saves it as an image in a specified directory. The subsequent thread executes object detection using tiny-YOLOv4. The system employs the watchdog package, an open-source Python API, to monitor file system events. The package aims to detect if new image files are retrieved from the camera thread. Once a new image is retrieved, it is queued for object detection.

### Performance evaluation metric

Our proposed RAViS framework is evaluated based on two key components, i.e. the ability to maintain resource availability (RAM, CPU, and storage) and object detection accuracy. The first component refers to the RAViS framework's ability to ensure sufficient resources are available for the object detection system. The second component refers to the ability of the object detection system to accurately identify and track objects of interest within the video stream with the adjustment made by the RAViS framework.

#### Resource availability

Let us consider *x* as the resource usage at time *i*, and n is the total point of time where each resource usage is recorded. Resources availability is measured based on the following aspects:

• Mean (Eq. (5)): The mean measures the data's central tendency. It indicates the average value of the data points and is affected by outliers.
  (5) $$\bar{x}=\;\frac{1}{n}\;\sum_{i=1}^{n}x_{i}$$
  *The mean of data indicates the data's central tendency.*

• Minimum (Eq. (6)) and maximum (Eq. (7)): These metrics give the data's range of values. They can be useful in identifying extreme values or outliers.
  (6) $$\min x_{1},\;x_{2},x_{3},\;\ldots x_{n}$$
  *The minimum value of a set of data.*
  (7) $$\max x_{1},\;x_{2},x_{3},\;\ldots x_{n}$$
  *The maximum value of a set of data.*

• Standard deviation (Eq. (8)): The standard deviation is another measure of the spread of the data. It indicates how much the data varies from the mean and is affected by outliers.
  (8) $$\text{std}=\;\sqrt{\frac{1}{n-1}\;\sum_{i=1}^{n}{\left(x_{i}-\;\bar{x}\right)}^{2}}$$
  *The standard deviation value of a set of data.*

• Quartile ranges (Eq. (9)): The IQR measures the data's spread. It indicates the range of values that cover the middle 50% of the data and is less sensitive to outliers than the standard deviation.
  (9) $$Q_{1}=\;x_{\left[n\;/\;4\right]}\;\&Q_{3}=\;x_{\left[3n\;/\;4\right]}$$
  *The Quartile ranges value of a set of data.*

By considering these metrics together, one can gain a more complete understanding of the distribution of the data, including its central tendency, range, and spread. For example, if the mean and median are similar and the standard deviation and IQR are low, it suggests that the data is tightly clustered around the center and there are few outliers. On the other hand, if the mean and median differ significantly and the standard deviation and IQR are high, it suggests that the data is more widely dispersed and may contain outliers.

#### Object detection accuracy

The impact of the RAViS framework on object detection accuracy is evaluated using Eq. (10). This metric indicates the True Positives (TP) ratio to the total number of detections. The TP indicates the correctness of detecting the presence of humans in an image, including partially correct detection (Eq. (11)). For example, three humans are in a room, but only two are detected. Then, it is still considered a correct detection. When no humans are detected, but humans (regardless of the number) are present, it is regarded as an incorrect detection.

(10) $$detAcc=\;\frac{TP}{\left(TP+TN+FP+FN\right)}$$

*Calculating the accuracy of object detection.*

(11) $$f\left(\text{TP}\right)\left\{ \begin{array}{cc} 1, & \text{if}\;\frac{n}{m}\;or\;\frac{n}{m}\;;n\;\text{is}\;\text{partially}\;\text{detected}\;\text{human}\;\text{with}\;n=\left[1,\;\left(m-1\right)\right]\;\text{and}\;m\;\text{is}\;\text{total}\;\text{detection}\\ 0, & \text{if}\;\text{otherwise} \end{array}\right.$$

*The function to map true positive to discrete values 1 or 0 based on the detection results*

Our RAViS framework is regarded as successful in managing resource consumption if the detection accuracy is improved or maintained while using fewer computational resources. On the other hand, if the detection accuracy is significantly reduced, it may indicate that the framework's adjustments were ineffective.

## Experimental results and analysis

The experiments conducted in the resource-aware framework included three scenarios: numeric data stream processing, video data stream processing without the RAViS framework, and video data stream processing with the RAViS framework. The aim was to observe the RAViS framework's robustness in different environments, i.e. in processing video data streams, and assess its impact on the system.

### Scenario 1: processing numeric data stream

This experiment scenario aimed to determine the baseline resource consumption required to process numeric data streams. The scenario involved simulating numeric data streams from a temperature sensor file. The data was generated by randomizing numbers between 24 and 34, with approximately 86,400 data streamed every second to the system. Results indicate that resource consumption was low, with an average RAM, CPU, and storage consumption of ±29.6%, ±6.35%, and ±57.2%, respectively, as presented in Table 1. These results suggest that processing such data requires minimal resources, making it a viable option for resource-limited environments.

**Table 1:** Sample of data records in Scenario 1.

Id	Time (H:M:s)	Time Elapsed (s)	RAM (%)	CPU (%)	Storage (%)
0	11:30:22	0.10	30.6	14.6	57.0
100	11:32:07	105.50	30.8	5.0	57.0
200	11:34:04	222.95	29.6	2.6	57.2
300	11:36:05	343.41	29.6	9.5	57.2
400	11:38:00	458.45	29.6	4.9	57.2
500	11:39:56	574.10	29.6	7.3	57.2
600	11:41:48	686.73	29.6	2.4	57.2
700	11:43:42	800.56	29.6	4.9	57.2
800	11:45:40	918.02	29.6	7.3	57.2
900	11:47:32	1030.65	29.6	5.0	57.2

### Scenario 2: processing video stream without ravis framework

In the second scenario, the system will function as intended without the RAViS framework. This scenario runs to determine how many resources would be required for this system without RAViS framework. Every second, an image will be taken by the system. As soon it's captured, it will be processed for object detection. After a few hours of operation, the system would have limited resources (Table 2).

**Table 2:** Sample of data records in Scenario 2.

Id	Time (H:M:s)	Time Elapsed (s)	RAM (%)	CPU (%)	Storage (%)
0	11:11:02	0.10	30.1	72.1	25.9
100	11:11:23	21.07	32.8	100	25.9
200	11:11:44	41.63	38.0	72.5	25.9
300	11:12:04	62.16	37.7	42.5	26.0
400	11:12:25	82.47	37.9	100	26.0
500	11:12:45	102.50	38.1	70.0	26.0
600	11:13:06	123.36	38.5	100	26.0
700	11:13:26	143.84	38.8	100	26.0
800	11:13:47	164.42	38.6	100	26.0
900	11:14:07	184.80	38.3	100	26.1

### Scenario 3: data stream with ravis framework

This scenario aims to observe the effect of RAViS framework on resource consumptions. The summary of resource consumption is presented in Table 3. Using the parameter sweeping technique, the RAViS framework was optimized to determine the optimum threshold. Threshold values for resource adaptation ranging from 40% to 60% for RAM and 60% to 80% for CPU were incrementally tested, and the optimal threshold was determined based on the lowest average of resource consumption (Table 4). The table shows that decreasing the lower threshold leads to decreased resource usage. The RAViS framework can notably lower the utilization of CPU by 40%. Similarly, RAM consumption can be reduced by 10%, whereas the reduction in storage consumption is 17%. These results show how much the RAViS framework has improved the resource efficiency to run the object detection system.

**Table 3:** Sample of data records in Scenario 3.

Id	Time (H:M:s)	Time Elapsed (s)	RAM (%)	CPU (%)	Storage (%)
0	05:42:07	0.10	30.2	82.2	42.5
100	05:42:29	21.43	33.3	33.3	42.5
200	05:42:50	42.68	37.5	90.2	42.6
300	05:43:11	63.93	38.1	30.0	42.6
400	05:43:32	84.37	38.8	62.2	42.6
500	05:43:52	104.72	38.8	72.5	42.6
600	05:44:13	125.59	38.8	97.4	42.7
700	05:44:33	146.22	38.8	38.1	42.7
800	05:44:54	166.65	38.6	7.5	42.7
900	05:45:15	187.48	39.0	100	42.7

**Table 4:** Average RAM, CPU and storage usage using different adaptation thresholds.

RAM	RAM Average Usage (%)	CPU	CPU Average Usage (%)	Storage	Storage Average Usage (%)
40	30.2	40	33.9	40	35.6
50	38.7	50	50.7	50	41.3
60	40.4	60	61.5	60	44.8
70	40.6	70	74.0	70	52.4

### Object detection performance

**Fig. 5:**
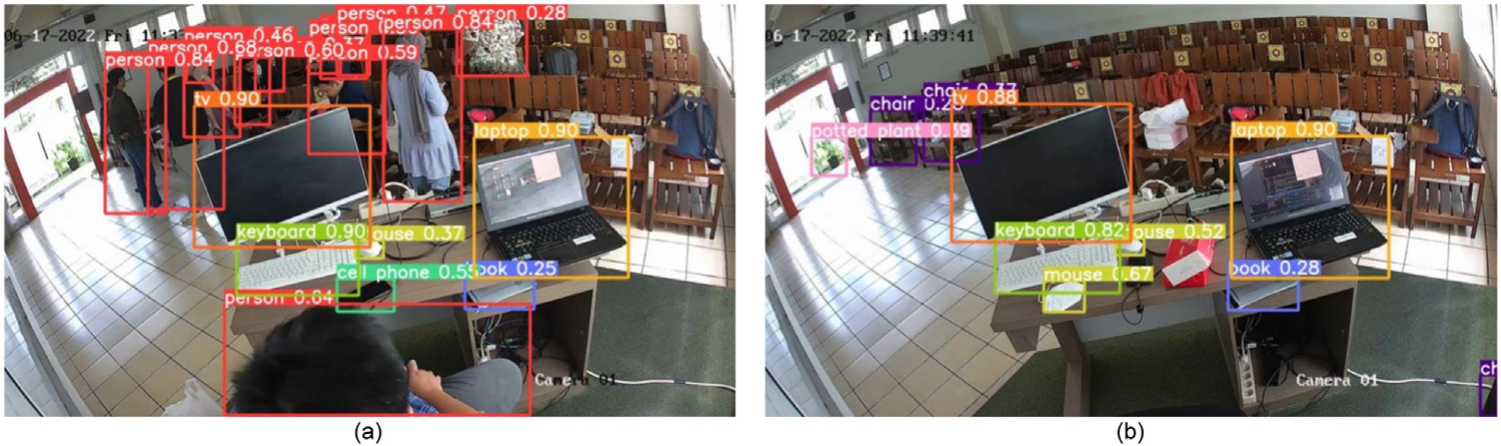
Object detection results (a) persons are detected in the room (b) no persons are detected.

We developed a simulation system to manage energy consumption in a room. Let us call the system the energy management system (EMS). By incorporating electrical equipment and objects in the room, EMS can issue an ON signal to maintain the electricity supply when it detects the presence of humans and an OFF signal when the room is empty to conserve energy. Our primary objective is to evaluate the effectiveness of our resource-aware object detection system using this EMS. Unlike motion sensors, the EMS accurately detects the presence of a human in a room, even if they are not moving. This ability ensures a more reliable and precise energy management process. When people are present, the electrical current is maintained (Fig. 5(a)), and when they are not detected, the electrical current is turned off (Fig. 5(b)). These results demonstrate the system's accuracy despite adjustments made by the RAViS framework to the system parameters.

Our proposed system has a detection accuracy of 99.21%, calculated by dividing the number of correctly detected video frames by the ground truth of the human observation (Eq. (10) and Eq. (11)). Of these, 43.65% were fully correct detections, and 56.34% were partially correct detections, indicating that the system could detect some but not all people in the room or detect them as other objects. Nevertheless, the system meets the requirements of the EMS since it accurately detects the presence or absence of people in a room.

### Experiment analysis

We have evaluated our resource-aware object detection system against conventional data stream models that process sensor-generated numerical data. Throughout the system's operation, we monitored resource consumption in various scenarios and analyzed the system's resource usage, including RAM, CPU and storage, based on the collected data. The RAViS framework's efficiency in resource consumption is attributed to its ability to process data while maintaining the computational load.

#### RAM

**Fig. 6:**
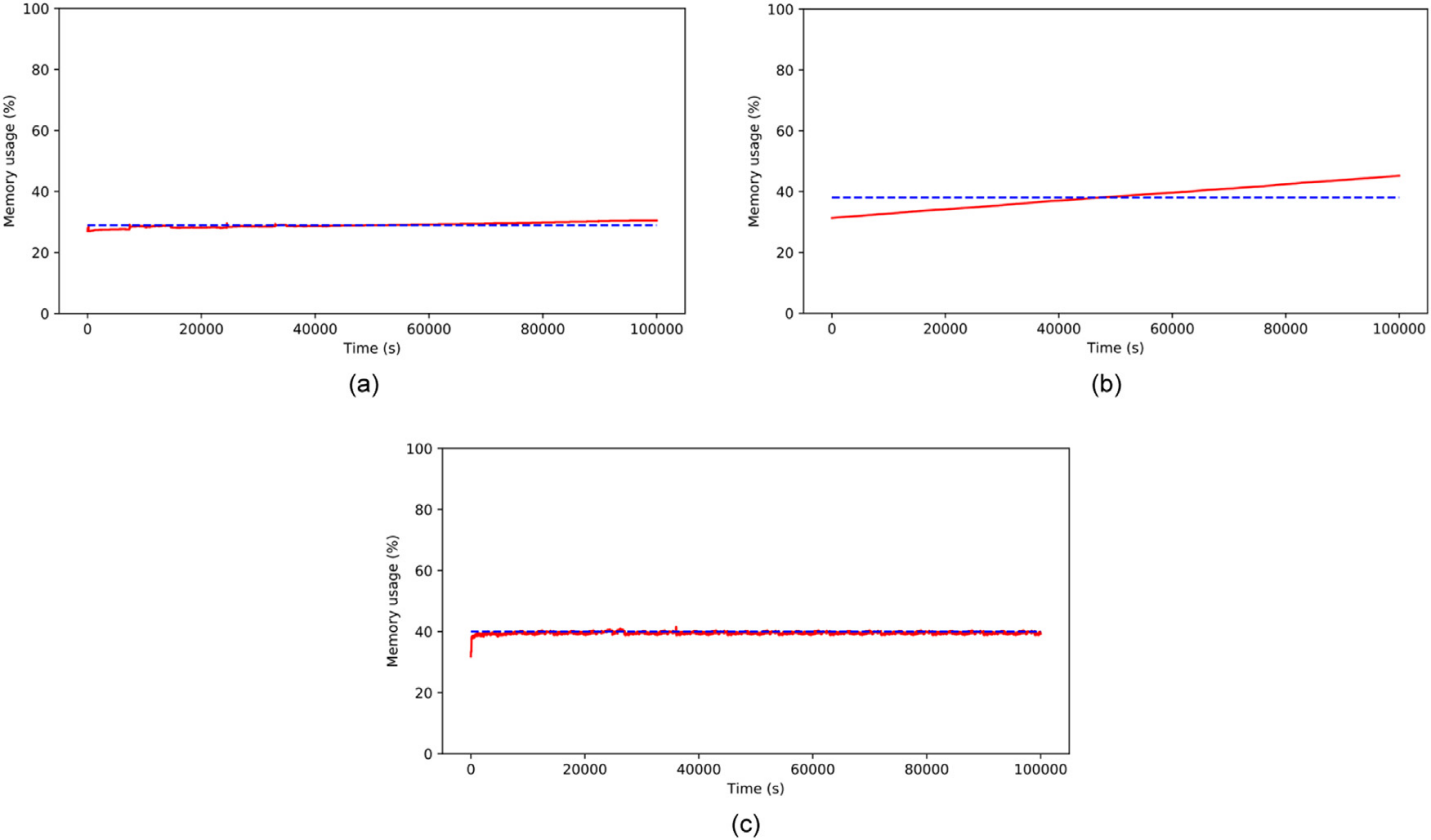
RAM usage on (a) numeric data streams (b) video streams without RAViS framework (c) video streams with RAViS framework.

RAM consumption is summarized in Table 5 while the while the time series of RAM consumption of each scenario are shown in (Fig. 6(a), (b), and (c)). RAM usage for storing numeric data in Scenario 1 remains low, peaking at only 31%, despite a gradual increase in the graph. This trend is due to the system's simple calculations that do not require extensive processing. The use of the RAViS platform has increased memory consumption by approximately 65%. However, the standard deviation decreased by 1.3, indicating that RAM consumption has become more consistent.

**Table 5:** RAM usage (%) in each scenario.

	RAM usage (%)		
	Scenario 1	Scenario 2	Scenario 3
mean	29.0	36.7	42.4
std	28.7	3.5	2.2
min	28.6	29.7	30.9
25%	29.0	33.6	40.4
50%	29.0	36.9	41.4
75%	29.0	39.5	44.6
max	31.0	44.8	47.8

Scenario 2 demonstrates a decline in RAM usage at the beginning of the experiment suggesting a delay between the observation start time and image capture and processing. This trend is likely due to a queue watchdog in the image processing thread using tiny YOLO. Consequently, RAM remains idle for a while. The image recording and processing timeline for object detection strengthens this finding.

Scenario 2 demonstrates a decline in RAM usage at the beginning of the experiment suggesting a delay between the observation start time and image capture and processing. This trend is likely due to a queue watchdog in the image processing thread using tiny YOLO. Consequently, RAM remains idle for a while. The image recording and processing timeline for object detection strengthens this finding.

The processing of data streams depends on the rate at which the data streams arrive. This dependency can cause a problem in the performance of a data stream processing system as such systems require immediate feedback to operate processes on limited resources. Our proposed system experiences the same issue. The longer our system runs, the longer it takes to detect objects. We have run the system for approximately 9 h. We have recorded that the average time required to detect objects increases significantly due to a piling queue watchdog in the image processing thread, reaching an average time of 8210 s or 136 min (Table 6).

**Table 6:** Scenario 2 image capturing and detection time difference.

Image Capturing (H:M:S)	Detection Process (H:M:S)	Time Difference (s)	Score
10:28:03	10:28:12	9	0.5
10:36:27	11:03:44	1637	0.53
11:13:52	11:22:45	533	0.77
11:17:59	11:40:55	1376	0.9
11:21:37	11:57:20	2143	0.48
11:24:31	12:10:10	2739	0.49
11:31:20	12:40:57	4177	0.46
11:37:11	13:07:27	5416	0.5
11:45:51	13:46:36	7245	0.58
11:51:22	14:10:48	8366	0.66

RAM usage in experiment Scenario 3 increased during the first 4–5 h due to longer image capturing times and faster object detection start times compared to Scenario 2. The time difference between Scenario 3 and Scenario 2 was faster with an average of 7.07 s (Table 7). The required time to process images is much less than that of in Scenario 2. RAM usage tended to decrease when it reached the threshold value. After 6 h, RAM usage decreased due to RAViS framework feedback, causing a delay in image capturing.

**Table 7:** Scenario 3 image capturing and detection time difference.

Image Capturing (H:M:S)	Detection Process (H:M:S)	Time Difference (s)	Score
18:16:41	18:16:50	9	0.72
18:31:47	18:31:54	7	0.50
18:55:00	18:55:13	13	0.61
20:08:06	20:08:10	4	0.66
22:18:41	22:18:48	7	0.99
22:41:18	22:41:24	6	0.88
22:56:50	22:56:57	7	0.47
23:29:10	23:29:15	5	0.50
00:09:47	00:09:51	4	0.65
01:10:58	01:11:09	11	0.56

#### CPU

**Fig. 7:**
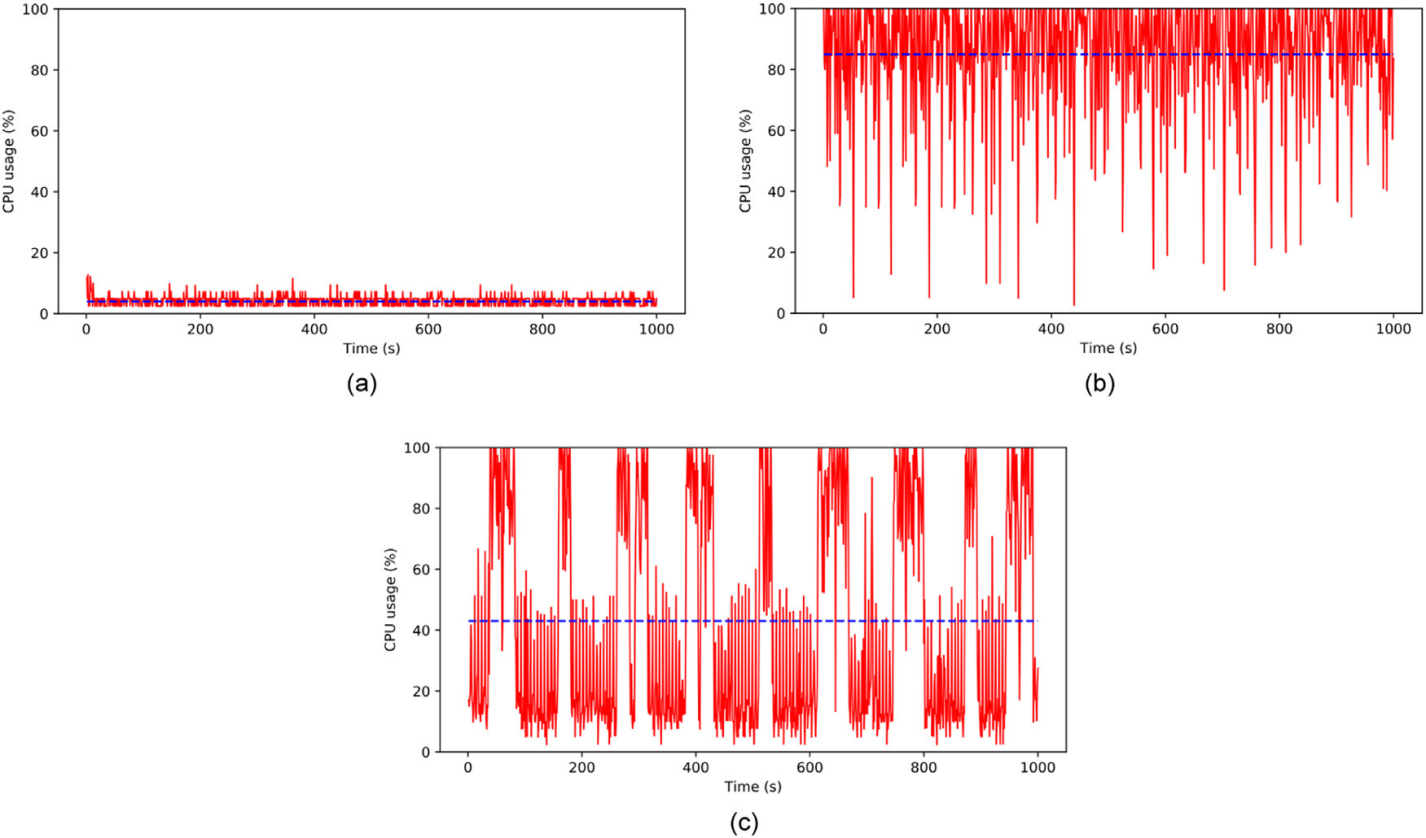
CPU usage on (a) numeric data streams (b) video streams without RAViS framework (c) video streams with RAViS framework.

The summary of CPU consumption is shown in Table 8, while the dynamics of CPU consumption are shown in Fig. 7(a), (b), and (c). We can see that the data set is evenly distributed around the mean, suggesting that the data set is fairly symmetrical. Thus, we can infer that CPU consumption in Scenario 1 is considerably low, with a 4.3% average, and the highest consumption is only 16.6%. This trend shows that mining numeric data does not require extensive resources.

**Table 8:** CPU usage (%) in each scenario.

	CPU usage (%)		
	Scenario 1	Scenario 2	Scenario 3
mean	4.2	84.3	47.9
std	2.3	19	36.1
min	2.3	2.4	2.4
25%	2.4	76.9	14.6
50%	2.6	90	30.8
75%	5	100	87.2
max	36.6	100	100

In Scenarios 2 and 3, color images are represented by pixels, each containing three scalar components representing red, blue, and green values. These components are stored in arrays of classes uint8 and uint16, with the former having a value range of 0–255 and stored in 8 bits. The number of pixels in an image increases with higher resolution, leading to more extensive image-forming arrays.

Scenario 2 produced a broad range of minimum and maximum values. The quartiles suggest that there may be some skewness toward higher values. Thus, we can say that Scenario 2 results in higher resource consumption with an average CPU usage of 84.3%, much higher than in Scenario 3, which only consumes 47.9% CPU usage. These trends are due to the RAViS framework adaptation in scenario 3, when resource utilization starts to reach the threshold.

Processing image data requires more computational resources than numeric data, as demonstrated by the higher CPU consumption observed in Scenarios 2 and 3 compared to Scenario 1. These findings provide insight into the resource requirements of image processing applications and can inform the development of more efficient data processing systems.

#### Storage

The storage usage varied depending on the images stored in each scenario, see Table 9. The graph indicated that the data depicted the storage usage while the system ran. The device had a capacity of 32GB, with approximately 24GB remaining, including operating system data and other data. The experiment showed the significant impact of stored images and the need to manage storage capacity effectively.

**Table 9:** Storage usage (%) in each scenario.

	Storage usage (%)		
	Scenario 1	Scenario 2	Scenario 3
mean	57.2	57.9	44.4
std	0.0085	18.5	7.3
min	57	25.9	25.8
25%	57.2	41.7	40.2
50%	57.2	57.8	43.9
75%	57.2	73.9	49.9
max	57.2	89.9	57.1

**Fig. 8:**
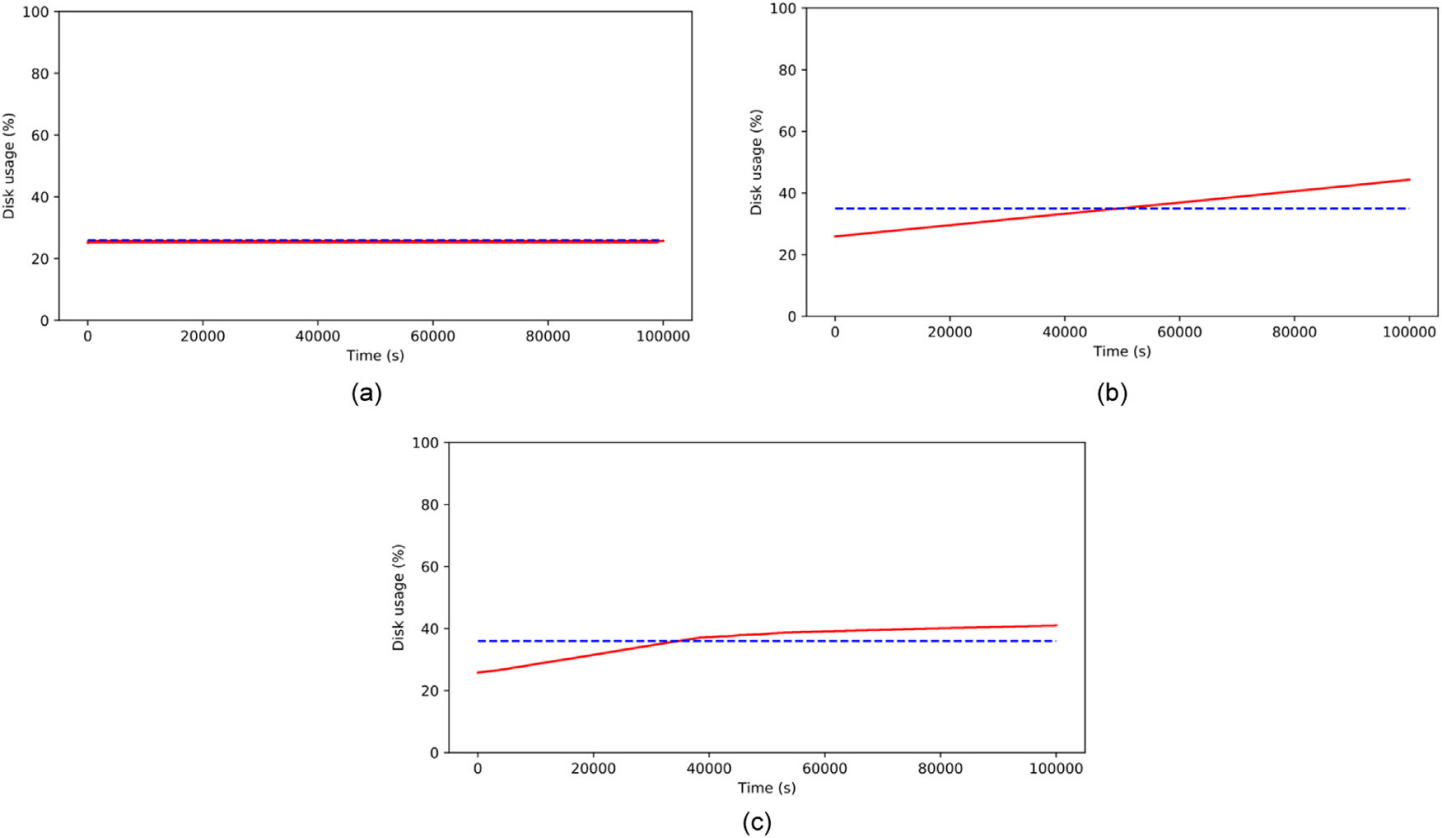
Storage usage on (a) numeric data streams (b) video streams without RAViS framework (c) video streams with RAViS framework.

Scenario 1 just has constant storage usage because no significant data is saved into the device (Fig. 8(a)). The record shows that the storage usage percentage rose by just 0.2%. This trend is caused by a cache file from the operating system. The experiment revealed that storage usage in Scenario 2 consistently increased (Fig. 8(b)). In contrast, Scenario 3 showed a gradual increase in storage usage after running the system for a few hours, as depicted in Fig. 8(c). This trend was because the storage usage in Scenario 3 reached the threshold value of 43%, causing a slower image-capturing time and reducing storage usage. Scenario 2 caused the storage usage to increase from 25.9% (before the detection system ran) to 89.9%, indicating a quicker resource consumption rate than Scenario 3, where the storage usage went from 25.8% to 57.1% in the same timeframe.

Experiment results indicate that the resource-aware framework ensures that resource availability can be maintained according to the threshold set. The purpose of safeguarding resource availability is to ensure that resources (RAM, CPU, and storage) remain available for the system, even on machines with limited resources. Simultaneously, the utilization of this framework does not significantly compromise the performance of object detection processes in a case study involving the presence or absence of individuals in a room.

## Conclusion

This study presents a novel system that elaborates a resource-aware framework and a deep learning-based object detection algorithm (called the RAViS framework) to detect objects from continuous images captured by IP cameras, considered data streams. The integration of the resource-aware framework aims to maintain resource availability during the object detection process, which may consume significant resources, unlike the ordinary data streams (numeric, text, etc.) produced by sensors or similar devices. The RAViS framework manages the RAM, CPU, and storage, where the CPU monitoring controls the interval of image processing, the RAM monitoring manages the interval of image capturing, and the storage monitoring manages the interval of freeing up storage.

We demonstrate that our system can maintain resource availability, thus guaranteeing that the object detection process runs continuously. In the future, we plan to extend our system to use devices with built-in GPUs, such as Nvidia Jetson, or using the Neural Compute Stick (NCS) by Intel to provide a virtual processing unit. This extension will reduce the processing load and make the overall process lighter than the current system.

## CRediT authorship contribution statement

**Ary Mazharuddin Shiddiqi:** Conceptualization, Methodology, Writing – original draft. **Edo Dwi Yogatama:** Software, Validation, Data curation. **Dini Adni Navastara:** Formal analysis, Validation.

## Declaration of Competing Interest

The authors declare that they have no known competing financial interests or personal relationships that could have appeared to influence the work reported in this paper.

## Data Availability

No data was used for the research described in the article.

## Appendix Supplementary materials

Image, application 1
