# Supplementary material for: Resource-aware video streaming (RAViS) framework for object detection system using deep learning algorithm
Source: MethodsX. 2023 Jul 15;11:102285. doi: 10.1016/j.mex.2023.102285 (PMC10391672; doi:10.1016/j.mex.2023.102285)
Supplement: Supplementary file 1 [file mmc1.docx]

**Appendix (Pseudo Code)**

Initialize ResourceThresholds // Set predefined thresholds for resource availability

function RAViS(objectDetectionAlgorithm, resourceAvailability):

if resourceAvailability < ResourceThresholds:

AdjustAlgorithmParameters(objectDetectionAlgorithm)

PerformObjectDetection(objectDetectionAlgorithm)

return DetectedObjects

function AdjustAlgorithmParameters(objectDetectionAlgorithm):

// Adjust algorithm parameters based on resource availability

// Implement logic to dynamically optimize resource usage

Input: ram = monitorRAMPercentage()

Input: cpu = monitorCPUPercentage()

cRAM = 1

cCPU = 1

cStorage = 1

while TRUE do

// PROCEDURE WatchRAM

if (ram > τRAM and ram < 100) then

ramWarning = FALSE

tcapture = 1 + (1 - τRAM) × cRAM

cRAM = cRAM + 1

else if (ram >= 100) then

ramWarning = TRUE

else

cRAM = 1

ramWarning = FALSE

end if

// PROCEDURE WatchCPU:

if (cpu > τCPU and cpu < 100) then

cpuWarning = FALSE

tprocess = randomBetween(1 - 5) × cCPU

cCPU = cCPU + 1

else if (cpu >= 100) then

cpuWarning = TRUE

else

cpuWarning = FALSE

cCPU = 1

end if

// PROCEDURE WatchStorage:

if (storage > τStorage and storage < 100) then

storageWarning = FALSE

treset = 1 + (1 - τStorage) × cStorage

cStorage = cStorage + 1

else if (storage >= 100) then

storageWarning = TRUE

else

storageWarning = FALSE

cStorage = 1

end if

end while

return UpdatedObjectDetectionAlgorithm

function PerformObjectDetection(objectDetectionAlgorithm):

def detect(self, image):

if notContinueProcess():

return 0

elif ContinueProcess():

try:

load image(image)

layer_names = getLayerNames()

output_layers = [layer_names[i - 1] for i in getUnconnectedOutLayers()]

outs = forward(output_layers)

define confidences = []

define class_ids = []

for out in outs:

for detection in out:

scores = detection

class_id = argmax(scores)

confidence = scores[class_id]

if confidence > YOLO_CONFI:

class_ids.append(class_id)

confidences.append(confidence)

DetectedObjects = getConfidenceAvg(confidences)

return DetectedObjects

except:

# Handle any exceptions that occur during the detection process

return None

def confiAvg(self, confidences):

if len(confidences) != 0:

return sum(confidences) / len(confidences)

return 0

def prepareImage(self, image):

if not stream:

image = imageRead(image)

if image is not None:

blob = blobFromImage(image, YOLO_SCALE, YOLO_IMGSIZE, (0, 0, 0), True, crop=False)

setInput(blob)

else:

print('[]\tYOLO image not read correctly')

def isContinueProcess(self):

if resumeTime is not None:

now = datetime.now()

diff = now - self.resumeTime

diff = diff.total_seconds()

if diff >= self.timeout:

return True

return False

def setTimeoutYOLO(self, num):

if resumeTime is None:

resumeTime = datetime.now()

self.timeout = num

print("[]\tYOLO process delay for {} s".format(num))
